# Glycyrrhetinic acid induces cytoprotective autophagy via the inositol-requiring enzyme 1α-c-Jun N-terminal kinase cascade in non-small cell lung cancer cells

**DOI:** 10.18632/oncotarget.6084

**Published:** 2015-11-03

**Authors:** Zheng-Hai Tang, Le-Le Zhang, Ting Li, Jia-Hong Lu, Dik-Lung Ma, Chung-Hang Leung, Xiu-Ping Chen, Hu-Lin Jiang, Yi-Tao Wang, Jin-Jian Lu

**Affiliations:** ^1^ State Key Laboratory of Quality Research in Chinese Medicine, Institute of Chinese Medical Sciences, University of Macau, Macao, China; ^2^ Department of Chemistry, Hong Kong Baptist University, Kowloon Tong, Hong Kong, China; ^3^ State Key Laboratory of Natural Medicines, Department of Pharmaceutics, China Pharmaceutical University, Nanjing, China

**Keywords:** glycyrrhetinic acid, autophagy, JNK, IRE1α, protective

## Abstract

Glycerrhetinic acid (GA), one of the main bioactive constituents of *Glycyrrhiza uralensis* Fisch, exerts anti-cancer effects on various cancer cells. We confirmed that GA inhibited cell proliferation and induced apoptosis in non-small cell lung cancer A549 and NCI-H1299 cells. GA also induced expression of autophagy marker phosphatidylethanolamine-modified microtubule-associated protein light-chain 3 (LC3-II) and punta formation of green fluorescent protein microtubule-associated protein light-chain 3. We further proved that expression of GA-increased autophagy marker was attributed to activation instead of suppression of autophagic flux. The c-jun N-terminal kinase (JNK) pathway was activated after incubation with GA. Pretreatment with the JNK inhibitor SP600125 or silencing of the JNK pathway by siRNA of JNK or c-jun decreased GA-induced autophagy. The endoplasmic reticulum (ER) stress responses were also apparently stimulated by GA by triggering the inositol-requiring enzyme 1α (IRE1α) pathway. The GA-induced JNK pathway activation and autophagy were decreased by IRE1α knockdown, and inhibition of autophagy or the JNK cascade increased GA-stimulated IRE1α expression. In addition, GA-induced cell proliferative inhibition and apoptosis were increased by inhibition of autophagy or the JNK pathway. Our study was the first to demonstrate that GA induces cytoprotective autophagy in non-small cell lung cancer cells by activating the IRE1α-JNK/c-jun pathway. The combined treatment of autophagy inhibitors markedly enhances the anti-neoplasmic activity of GA. Such combination shows potential as a strategy for GA or GA-contained prescriptions in cancer therapy.

## INTRODUCTION

Non-small cell lung cancer (NSCLC) is one of the most common cancers and a leading cause of cancer death worldwide [1]. The survival rate remains very poor and the treatment cost is very high despite various methods for NSCLC treatment, including surgical resection, chemotherapy, radiotherapy, and small molecular target therapy [2]. Except for upper treatment strategies, traditional Chinese prescriptions are also widely used for NSCLC treatment in clinical medicine in China because of their efficient therapeutic effect and low cost [3, 4]. Prescriptions used for lung cancer therapy in China include “Jie-Geng Decoction,” “Gan-Cao-Gan-Jiang Decoction,” and “Bai-He-Gu-Jin Decoction,” which mainly contain *Glycyrrhiza uralensis* Fisch [5, 6]. Glycyrrhetic acid (GA), one of the primary components and bioactivity compounds of *G. uralensis* Fisch *in vivo*, has been demonstrated to exhibit various pharmacological anti-cancer, hepatoprotective, and anti-inflammatory properties [7–9]. The anti-cancer property of GA has been examined in several types of cancer cells, including breast cancer MCF-7 and MDA-MB-231 cells, gastric cancer BGC823 and SGC7901 cells, as well as prostate cancer LNCaP cells. The potential mechanisms of the anti-cancer properties of GA involve reduction of proliferation, induction of apoptosis and cell cycle arrest, as well as inhibition of cellular metastasis [9–11]. GA also prevents tumor initiation and exhibits remarkable anti-growth and anti-metastasis properties *in vivo* without causing side effects [11–13].

Autophagy is a conserved metabolic pathway that clears and recycles damaged proteins or organelles in a lysosome-dependent manner for cell survival [14, 15]. The process begins when phagophores emerge and nucleate at the phagophore assembly site. Phagosomes elongate to form autophagosomes via two ubiquitination-like systems, namely, the phosphatidylethanolamine-modified microtubule-associated protein light-chain 3 (LC3-II) system and the autophagy-related protein ATG12-ATG5-ATG16 system. Autophagosomes then fuse with lysosomes to form autolysosomes and degrade their cargo [16–19]. A number of studies indicate that autophagy is stimulated under starvation and hypoxia through various tumor cell survival mechanisms and that inhibition of autophagy obviously decreases tumor growth [20, 21]. In addition, after chemotherapeutic drug treatment, the autophagy level of tumor cells increases to enhance drug resistance and decrease the anti-cancer effects of chemotherapeutics [22, 23]. Therefore, targeting autophagy to enhance the therapeutic effects of anti-cancer agents presents a novel approach for tumor therapy.

The Akt/mammalian target of rapamycin (mTOR) is identified as the main and classical pathway for autophagy activation. Inhibition of the Akt/mTOR cascade apparently increases autophagy. Rapamycin, a well-known mTOR inhibitor, is widely used as an autophagy inducer [24–26]. The mitogen-activated protein kinase family is also an important mediator of autophagy. Our previous studies demonstrate that activation of extracellular signal-regulated kinase (ERK) by diverse compounds can induce autophagy [27–29]. C-Jun N-terminal kinase (JNK) further plays a key role in endoplasmic reticulum (ER) stress-induced autophagy. In JNK pathway-deficient *in vitro* and *in vivo* models, ER stress-induced cell death is remarkably enhanced in the absence of autophagy [30, 31].

In this study, we confirmed that GA induces cytoprotective autophagy in NSCLC A549 and NCI-H1299 cells by IRE1α-JNK/c-jun cascade activation and that inhibition of autophagy or the JNK pathway increases GA-induced inhibitory effects and apoptosis.

## RESULTS

### GA induces cell proliferative inhibition, apoptosis, and autophagy in A549 and NCI-H1299 cells

**Figure 1:**
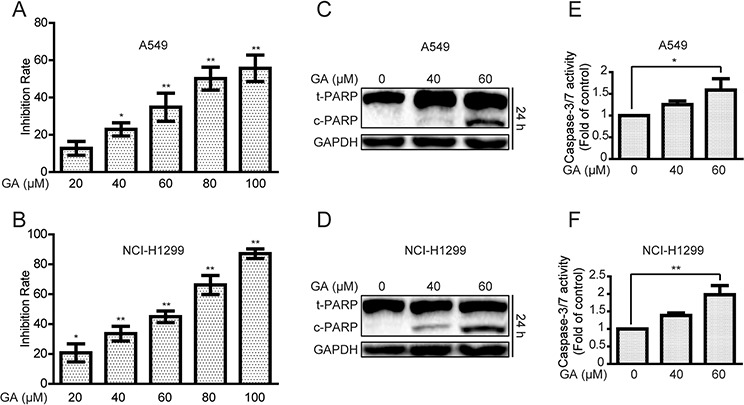
GA increases cell proliferative inhibition and apoptosis in A549 and NCI-H1299 cells **A–B.** Cells were treated with different concentrations of GA for 24 h. The anti-proliferative effects of GA on A549 and NCI-H1299 cells were evaluated by MTT assay. *P < 0.05 and **P < 0.01, compared with the 0 μM GA treatment (control). **C–D.** After treatment with GA for 24 h, A549 and NCI-H1299 cells were analyzed to determine indicated changes in proteins by Western blot analysis. **E–F.** A549 and NCI-H1299 cells were treated with GA for 24 h, and the activation of caspase-3/7 was determined using a commercial assay kit. *P < 0.05 and **P < 0.01.

We initially investigated the effects of GA on A549 and NCI-H1299 cells proliferation. As shown in Figure 1A–1B, GA remarkably increased inhibition rates in a concentration-dependent manner. The colony formation ability of A549 was decreased after GA treatment (Supplementary Figure S1A). The protein expressions of cleaved poly (ADP-ribose) polymerase (PARP), a biomarker of apoptosis [32], and caspase-3/7 activation were detected. GA increased cleaved PARP expression and caspase-3/7 activation (Figure 1C–1F and Supplementary Figure S1B). In addition, annexin V-FITC and propidium iodide dual labeling indicated that exposure of A549 cells to GA increased apoptotic cell percentages (Supplementary Figure S1C). Apoptotic chromatin condensation and DNA fragmentation were also observed after GA treatment by Hoechst 33342 staining assay (Supplementary Figure S1D). These data suggested that GA induced apoptosis in NSCLC A549 and NCI-H1299 cells.

**Figure 2:**
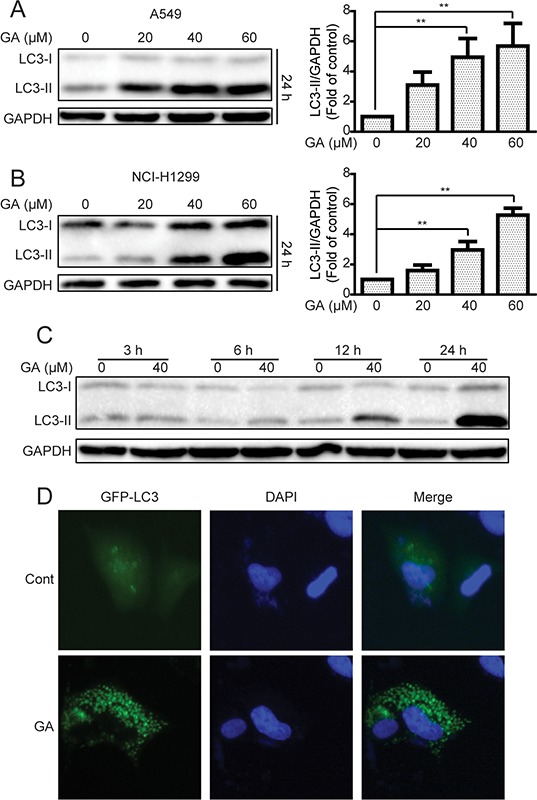
GA increases LC3-II expression and GFP-LC3 punta formation in A549 and NCI-H1299 cells **A–B.** A549 and NCI-H1299 cells were treated with various concentrations of GA for 24 h, and cell extracts were analyzed to determine changes in protein expression by Western blot analysis. *P < 0.05 and **P < 0.01. **C.** A549 cells were treated with GA (40 μM) for the indicated time. Western blot was used to detect protein expression. **D.** A549 cells were transiently transfected with the GFP-LC3 plasmid with or without GA (40 μM) for 24 h. GFP-LC3 punta formation was determined using In Cell Analyzer 2000, and typical images were presented.

We then determined whether or not GA could induce autophagy in NSCLC A549 and NCI-H1299 cells. Western blot assay was performed to examine the protein expression of LC3-II, which is essential for autophagy formation and mainly used as a protein marker of this phenomenon [33]. LC3-II protein expression in NSCLC cells increased in a concentration- and time-dependent manner after GA treatment compared with that in the control group (Figure 2A–2C and Supplementary Figure S2A). An immunoreactive band corresponding to processed LC3-II was slightly enhanced as early as 6 h after GA (40 μM) treatment. This band intensified with prolonged treatment, particularly after a 24 h treatment (Figure 2C and Supplementary Figure S2A). Green fluorescent protein (GFP)-LC3 plasmid was further transfected into A549 cells to characterize GA-induced autophagy and observed diffused localization of GFP-LC3 in the control cells. By comparison, a 24 h cell exposure to GA (40 μM) resulted in extensive GFP-LC3 punta generation (Figure 2D). In GFP-LC3 stable expression HeLa cells. More GFP-LC3 punta were generated in the GA-treated group than in the control group (Supplementary Figure S2B). These data indicated that GA increased autophagy marker expression in cancer cells.

### GA induces autophagic flux in A549 and NCI-H1299 cells

**Figure 3:**
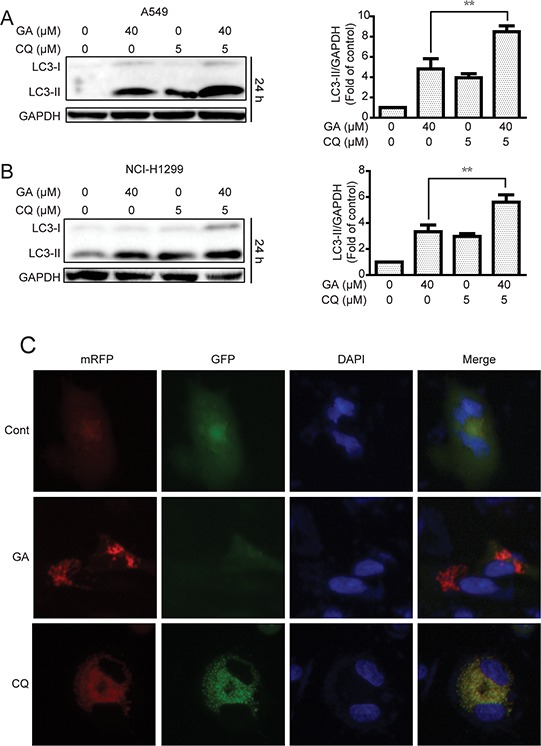
GA induces autophagic flux in A549 and NCI-H1299 cells **A–B.** A549 and NCI-H1299 cells were cultured in GA (40 μM) for 24 h with or without CQ pretreatment (10 μM, 1 h). Cell extracts were analyzed for protein expression using Western blot analysis. **C.** A549 cells were transiently transfected with the mRFP-GFP-LC3 plasmid for about 24 h and then treated with GA (40 μM) or CQ (5 μM) for 24 h. Cells were observed for changes in green and red fluorescence using In Cell Analyzer 2000, and typical images were shown.

**Figure 4:**
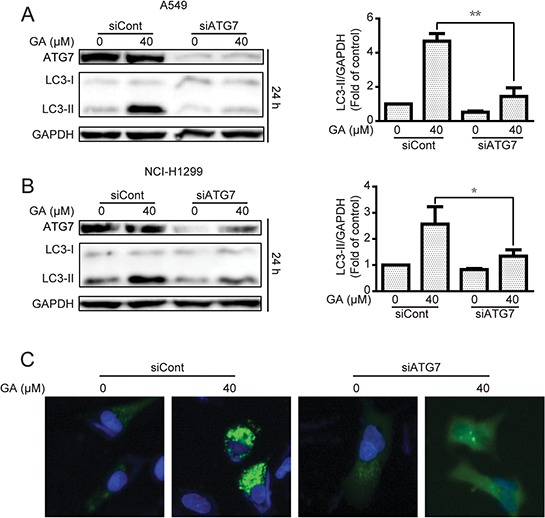
GA-induced autophagy is correlated with ATG7 in A549 and NCI-H1299 cells **A–B.** After transient transfection with scramble or ATG7 siRNA for 24 h, A549 and NCI-H1299 cells were treated with GA (40 μM) for 24 h. The indicated protein expression was evaluated by Western blot analysis. *P < 0.05 and **P < 0.01. **C.** A549 cells were transiently transfected with GFP-LC3 for 24 h and ATG7 siRNA for 24 h. Cells were then treated with GA (40 μM) for 24 h. GFP-LC3 punta were examined by In cell Analyzer 2000, and typical images were presented.

Increase in LC3-II protein or GFP-LC3 punta responses to the agent may increase the autophagic flux and/or suppress autolysosome degradation [33, 34]. To determine whether or not GA induced autophagic flux in NSCLC A549 and NCI-H1299 cells, cells were pre-treated with chloroquine (CQ), which affects lysosomal acidification to inhibit autophagy [33]. Results suggested that GA increased the protein expression of LC3-II when combined with CQ treatment (Figure 3A–3B), indicating that GA induced autophagic flux. These results were verified by transfecting cells with the mRFP-GFP-LC3 plasmid to evaluate the autophagic flux. mRFP-GFP-LC3 is used to examine induction or suppression of autophagic flux [33]. If autophagic flux increases and autolysosome maturation occurs normally, more red punta are observed in the cells because of the easy-to-quench GFP and the relatively acid-stable mRFP. Inhibition of autophagosome-lysosome fusion and/or lysosomal function lead to most punta exhibiting both red and green fluorescence; these punta appear yellow in merged images [35]. As shown in Figure 3C, large amounts of red-only punta were observed in GA-treated cells transfected with mRFP-GFP-LC3 plasmid, indicating that GA induced autophagic flux. Silencing of key autophagy factors, including ATG7, Beclin1, and ATG5, was performed to analyze GA-induced autophagic flux. Results showed that cells transfected with the siRNA of ATG7, Beclin1, or ATG5 exhibited lower levels of LC3-II accumulation after GA treatment, compared with that in the random siRNA control (Figure 4A–4B, Supplementary Figure S3A–S3B, and Supplementary Figure S4A–S4B). GA-induced GFP-LC3 punta were also reversed after genetic inhibition of autophagy by ATG7 knockdown (Figure 4C).

### GA induces autophagy by JNK/c-jun pathway activation in A549 and NCI-H1299 cells

**Figure 5:**
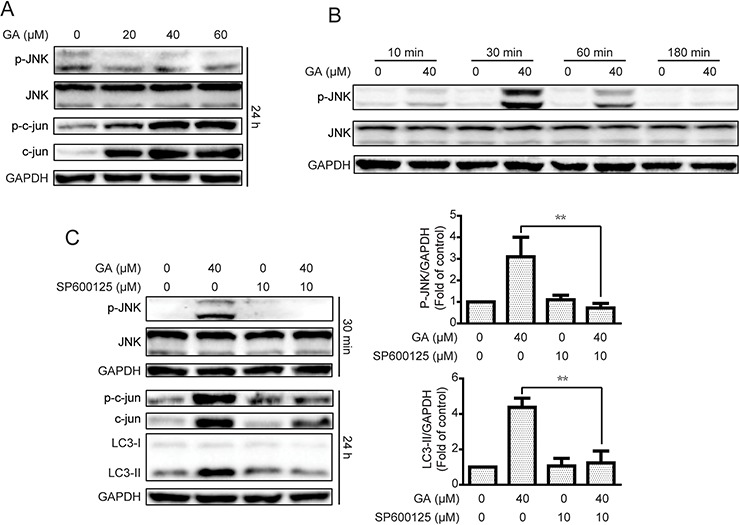
GA-induced autophagy is correlated with the JNK/c-jun pathway **A.** After treatment with GA for 24 h, the protein expression of A549 cells was evaluated by Western blot analysis. **B.** A549 cells were cultured with GA (40 μM) for specific periods. Western blot assay was performed to examine protein expression. **C.** A549 cells were treated with GA (40 μM) for 30 min or 24 h with or without the JNK pathway inhibitor SP600125 (10 μM, 1 h). Protein expression levels were detected by Western blot analysis. *P < 0.05 and **P < 0.01.

The JNK/c-jun pathway plays a pivotal effect in autophagy formation, and activation of JNK/c-jun can induce autophagy under diverse conditions [36, 37]. Therefore, we examined whether GA induced autophagy via JNK/c-jun cascade. As shown in Figure 5A and Supplementary Figure S5A, treatment of the indicated concentrations of GA for 24 h increased the expression of phosphorylated c-jun and total c-jun in A549 cells. However, the phosphorylated JNK was not increased after 24 h of GA treatment. Results suggested that JNK can be activated immediately when treated with certain compound and decline thereafter [38]. Therefore, phosphorylated JNK expression was investigated after various periods of GA treatment. JNK activation was observed after 30 min of GA exposure, and activation of kinase disappeared after 3 h; these findings were similar to our initial hypothesis (Figure 5B and Supplementary Figure S5B).

**Figure 6:**
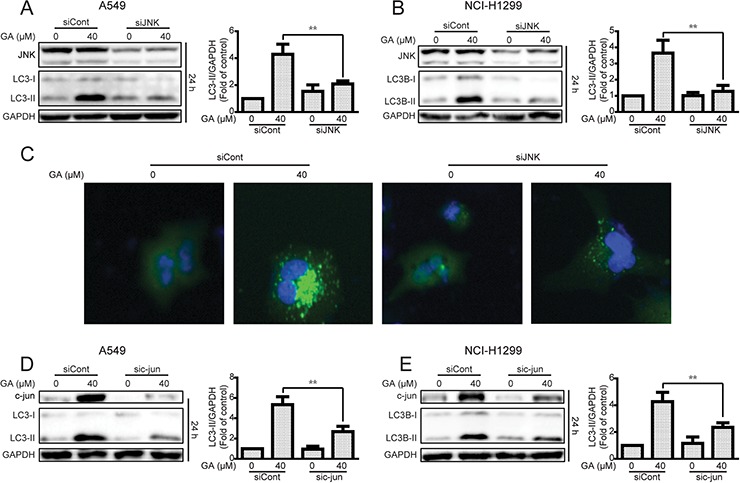
Knockdown of JNK or c-jun decreases GA-induced autophagy in A549 and NCI-H1299 cells **A–B**. A549 and NCI-H1299 cells were transiently transfected JNK siRNA, cultured for another 24 h, and treated with GA (40 μM) for 24 h. Western blot analysis was used to evaluate protein expression. *P < 0.05 and **P < 0.01. **C.** A549 cells were transiently transfected with GFP-LC3 for 24 h and JNK siRNA for 24 h. GA (40 μM) was added to the cells, and culture for another 24 h was performed. GFP-LC3 punta were examined using IN cell Analyzer 2000, and typical images were shown. **D–E.** After knockdown of c-jun by c-jun siRNA, A549 and NCI-H1299 cells were treated with GA (40 μM) for 30 min or 24 h. Western blot assay was used to detect changes in protein levels. *P* < 0.05 and **P < 0.01.

The JNK/c-jun pathway inhibitor SP600125 was used to examine whether GA induced autophagy by JNK/c-jun activation [39]. As shown in Figure 5C and Supplementary Figure S5C, GA-activated JNK and c-jun were decreased after pretreatment with SP600125 (10 μM, 1 h). The autophagy marker LC3-II induced by GA was also reversed, suggesting that GA induced autophagy via the JNK/c-jun pathway. To confirm the significance of JNK in GA-induced autophagy, siRNA was used for JNK silencing. Silencing of JNK by siRNA decreased the GA-induced LC3-II expression in both A549 and NCI-H1299 cell lines (Figure 6A–6B and Supplementary Figure S6A–S6B). The GA-induced GFP-LC3 punta were also suppressed after JNK knockdown in A549 cells (Figure 6C). In addition, c-Jun knockdown was performed to evaluate the effects of the JNK/c-jun pathway on GA-induced autophagy. Silencing of c-jun decreased GA-induced autophagy in A549 and NCI-H1299 cells, as evidenced by the reversal of GA-induced LC3-II expression (Figure 6D–6E and Supplementary Figure S6C–S6D). Thus, activation of the JNK/c-jun cascade was pivotal for GA-induced autophagy in NSCLC A549 and NCI-H1299 cells.

### ER stress-stimulated JNK/c-jun activation is critical for GA-induced autophagy in A549 and NCI-H1299 cells

**Figure 7:**
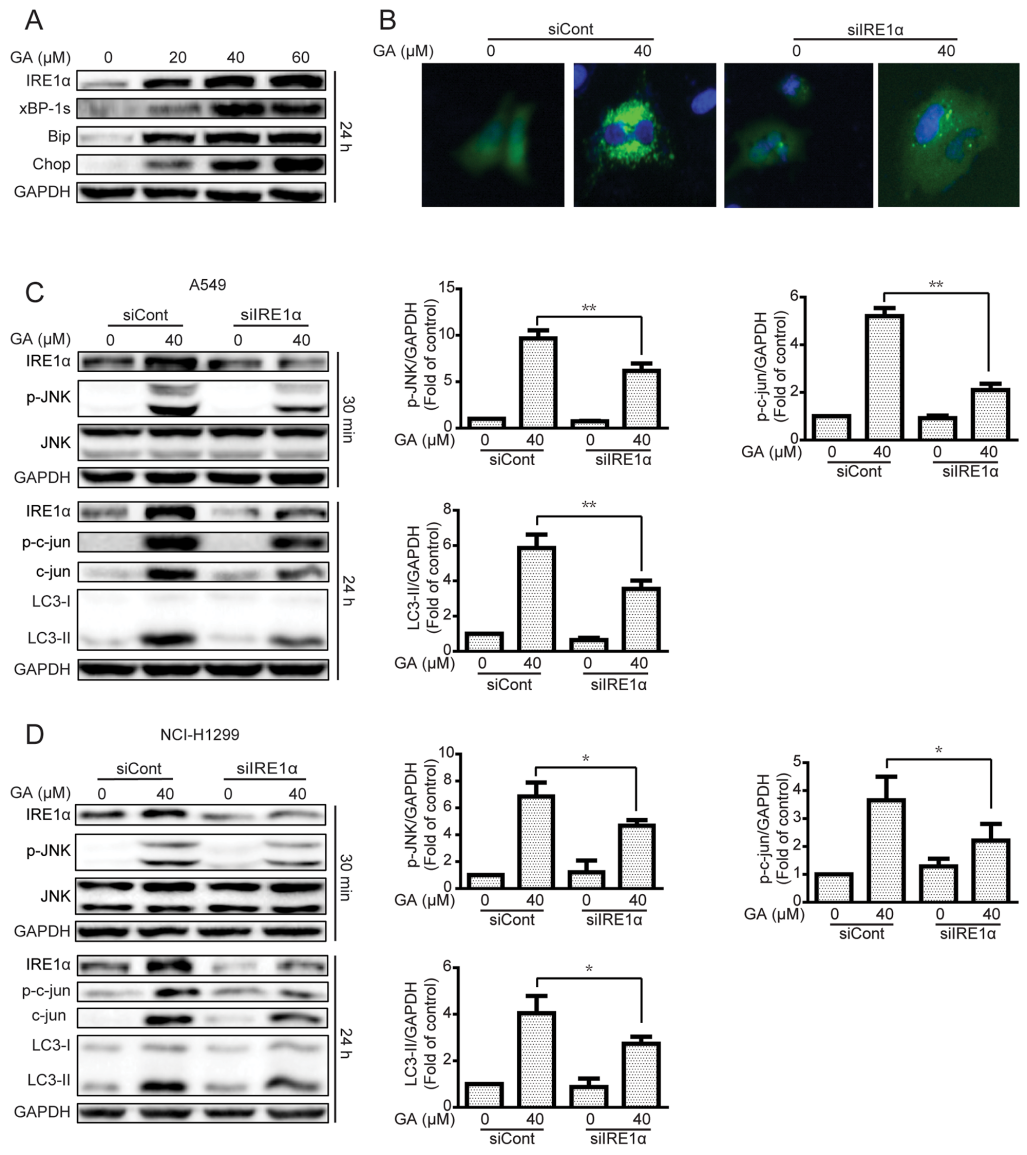
The IRE1α-JNK/c-jun pathway is essential for GA-induced autophagy in A549 and NCI-H1299 cells **A.** A549 cells were treated with varying concentrations of GA for 24 h, and the ER stress-related protein expression was evaluated by Western blot analysis. **B.** A549 cells were transiently transfected with GFP-LC3 and IRE1α siRNA for 24 h. Cells were treated with GA (40 μM) for 24 h. GFP-LC3 punta were evaluated by IN Cell Analyzer 2000, and typical images were shown. **C–D.** A549 and NCI-H1299 cells were infected with scramble or IRE1α siRNA and then incubated for 24 h. GA (40 μM) was added to the cells for 30 min or 24 h. Protein expression related to the JNK/c-jun pathway and autophagy was detected by Western blot analysis. *P* < 0.05 and **P < 0.01.

**Figure 8:**
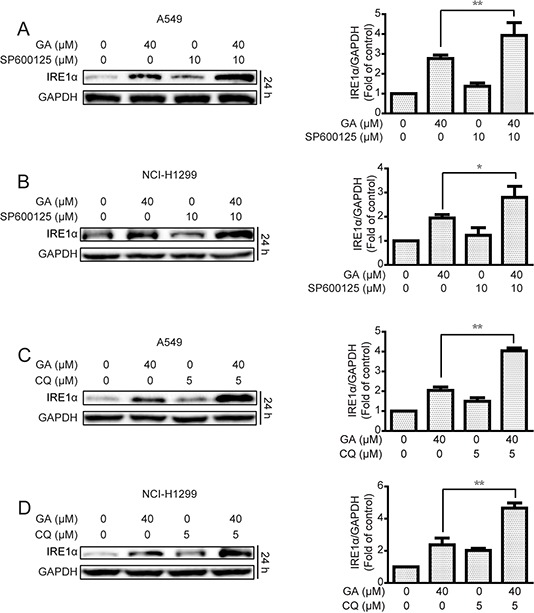
Inhibition of JNK/c-jun and autophagy increase GA-induced ER stress in A549 and NCI-H1299 cells **A–B.** After suppression of the JNK/c-jun cascade by SP600125 (10 μM, 1 h) pretreatment, A549 and NCI-H1299 cells were treated with GA (40 μM) for 24 h. Protein expression levels were detected by Western blot analysis. *P* < 0.05 and **P < 0.01. **C–D.** A549 and NCI-H1299 cells were treated with GA (40 μM) for 24 h with or without pretreatment of CQ (5 μM, 1 h). Western blot analysis was used to examine protein expression. *P* < 0.05 and **P < 0.01.

Induction of ER stress by two classical ER stress inducers, *i.e*., thapsigargin and tunicamycin, can stimulate autophagy [30, 31]. In the current study, GA induction of JNK/c-jun activation by ER stress stimulation was verified. Results showed that the expressions of the ER stress sensor IRE1α, its down-stream XBP-1s, and ER stress-related protein Chop and Bip were increased after GA treatment regardless of GA concentrations (20 μM) (Figure 7A and Supplementary Figure S7A). IRE1α knockdown by siRNA decreased GA-induced autophagy, as shown by decreased GFP-LC3 punta formation and LC3-II expression (Figure 7B–7D and Supplementary Figure S7B–S7C). In addition, the correlation between ER stress and JNK/c-jun in A549 and NCI-H1299 cells was determined. Knockdown of the ER stress sensor IRE1α partly inhibited the phosphorylation of JNK and c-jun after 30 min and 24 h of GA treatment, respectively (Figure 7C–7D and Supplementary Figure S7B–S7C). However, inhibition of the JNK/c-jun pathway by SP600125 or siRNA of c-jun increased GA-induced ER stress in A549 and NCI-H1299 cells (Figure 8A–8B and Supplementary Figure S8A), which may be attributed to the inhibition of GA-induced autophagy. Combined treatment of CQ or ATG7 knockdown also increased GA-induced ER stress (Figure 8C–8D and S8B), indicating that the autophagy inhibition could increase the cellular content of misfolded and damaged proteins, followed by ER stress stimulation [31]. The overall result indicates that GA induced autophagy in NSCLC A549 and NCI-H1299 cells by stimulation of IRE1α-JNK/c-jun pathway and inhibition of JNK/c-jun or autophagy increased GA-induced ER stress.

### Inhibition of autophagy and JNK increases GA-induced proliferative inhibition and apoptosis in A549 and NCI-H1299 cells

**Figure 9:**
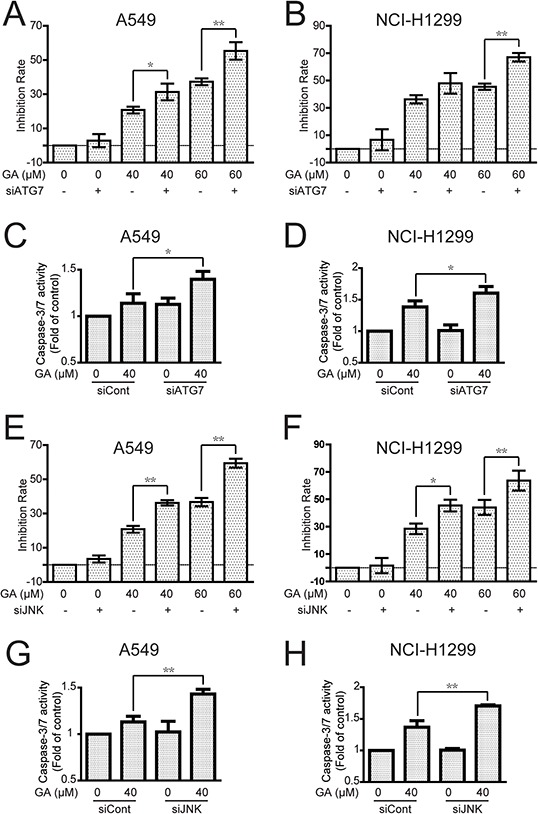
Inhibition of autophagy or JNK enhances GA-induced cell proliferative inhibition and apoptosis in A549 and NCI-H1299 cells **A–D.** A549 and NCI-H1299 cells were treated with GA for 24 h with or without pretreatment of ATG7 siRNA. Cell viability was evaluated using MTT assay, and caspase-3/7 activation was detected using a commercial assay kit. *P* < 0.05 and **P < 0.01. **E–H.** After knock down of JNK by siRNA, A549 and NCI-H1299 cells were incubated with indicated concentrations of GA for 24 h. Cell viability was evaluated by MTT assay, and caspase-3/7 activation was detected using a commercial assay kit. *P* < 0.05 and **P < 0.01.

The effects of autophagy (prosurvival or prodeath) in cancer therapy remain complex and inconclusive [40]. In the current study, GA-induced inhibition rate and caspase-3/7 activation were increased in both A549 and NCI-H1299 cell lines after suppression of autophagy by ATG7 siRNA or pretreatment with CQ (5 μM, 1 h) (Figure 9A–9D and Supplementary Figure S9A–S9D). Western blot analysis also indicated that the expression of cleaved PARP increased more extensively after autophagy inhibition (Supplementary Figure S9E–S9F). Given the significance of the IRE1α-JNK/c-jun pathway for GA-induced autophagy, we primarily evaluated the GA-induced inhibition rate and apoptosis by JNK/c-jun pathway suppression. As expected, the GA-induced inhibition rate and apoptosis were apparently increased after JNK/c-jun blockage in both A549 and NCI-H1299 cell lines (Figure 9E–9H and Supplementary Figure S9G). However, GA-induced inhibition rate and caspase-3/7 activation did not markedly change after IRE1α siRNA (Supplementary Figure S10A–S10D), which could be attributed to IRE1α induction not only of autophagy but apoptosis as well [41]. The overall data suggested that the GA-induced autophagy in NSCLC A549 and NCI-H1299 cells were cytoprotective.

## DISCUSSION

*Glycyrrhiza uralensis* Fisch is the primary ingredient of many traditional Chinese prescriptions for lung cancer therapy in China [42, 43], with triterpene saponin GA as its main metabolite and bioactive component *in vivo* [44, 45]. A number of studies show that GA can impart protection against tumorigenesis by inhibiting tumor growth and metastasis [11–13]. However, the present study confirms that GA stimulates apoptosis and cytoprotective autophagy in NSCLC A549 and NCI-H1299 cells. GA induced autophagy mainly by the IRE1α-JNK/c-jun pathway activation, and autophagy or JNK inhibition apparently increased GA-induced cell proliferative inhibition and apoptosis.

Several agents exhibit autophagic regulatory effects by increasing the levels of LC3-II or autophagosomes [46, 47]. However, increases in LC3-II expression or autophagosome accumulation may be an autophagy inducer (*i.e*., increases the autophagic flux) or inhibitor (*i.e*., inhibits the function of lysosome or the fusion of autophagosome with lysosome). Decreased LC3-II or autophagosomes may also be an autophagy inducer (*i.e*., accelerates autolysosome degradation) or inhibitor (*i.e*., inhibits the key components of autophagosome formation) [33, 34]. Methods such as combined treatment with CQ or use of the mRFP-GFP-LC3 plasmid must be considered to detect this property [33]. In the present study, the expression of LC3-II in combined GA and CQ treatment is more apparent than in either GA or CQ alone (Figure 3A–3B). More red fluorescent punta were observed in the GA-treated group than in the CQ-treated group (Figure 3C), indicating that GA induced autophagic flux in A549 and NCI-H1299 cells. In addition, compounds induce or inhibit autophagy depending on the cell line studied. For instance, paclitaxel, inhibits autophagic flux in breast cancer cells but stimulates autophagic flux in renal cancer cells [48, 49]. The current study suggests that GA-induced autophagy is universal, at least in NSCLC and hepatocellular carcinoma cells [28].

The mechanism of GA-induced autophagy was further investigated. In hepatocellular carcinoma cells, GA induces autophagy by ERK activation [28]. In the present study, GA also triggered ERK activation in NSCLC cells (Supplementary Figure S11A). However, inhibition of the ERK pathway by pre-treated with PD98059 or ERK siRNA could not decrease GA-induced LC3-II expression (Supplementary Figure S11B–S11C). The classical autophagy negative regulation cascade Akt/mTOR was further examined [24, 50], and results showed that GA could not decrease the activation of Akt/mTOR pathway-related proteins (Supplementary Figure S12A). Therefore, the mechanism of agent-induced autophagy was correlated with the cell type. We also observed this phenomenon in baicalein, one of the key flavones isolated from *Radix Scutellariae*. Baicalein induces autophagy in HepG2 cells by inhibition of the Akt/mTOR cascade [51]. Meanwhile, ERK pathway activation is critical for baicalein-induced autophagy in ovarian cancer HEY cells instead of Akt/mTOR cascade (data not shown). In the present study, GA-increased autophagy occurred mainly by the activation of IRE1α-JNK/c-jun cascade in NSCLC A549 and NCI-H1299 cells (Figures 5, 6, 7 and Supplementary Figures S5–S7). Activation of JNK was shown to induce the phosphorylation of Bcl-2, disruption of Bcl-2/Beclin1 complex, and release of Beclin1 to induce autophagy [52]. However, GA-induced autophagy by JNK activation is not correlated with the Bcl-2/Beclin1 complex (Supplementary Figure S13). Inhibition of autophagy or JNK/c-jun cascade sharply increased GA-induced ER stress (Figure 8 and Supplementary Figure S8), which may be attributed to the ER stress caused by the accumulation of unfolded proteins and autophagy being a metabolic pathway that clears misfolded and damaged proteins [14, 53].

The effects of autophagy (prosurvival or prodeath) depend on stimulators and cell types [54]. For instance, inhibition of autophagy increases the anti-hepatocellular carcinoma effects of linifanib but decreases the anti-rhabdomyosarcoma effects of piperlongumine [22, 40, 55]. The cytotoxicity of gefitinib to glioma cells mainly contributes to autophagy, whereas autophagy induced by gefitinib in lung cancer cells is cytoprotective [56, 57]. In the current study, inhibition of GA-induced autophagy or JNK/c-jun pathway remarkably increased GA-induced cell proliferative inhibition and apoptosis (Figure 9 and Supplementary Figure S9), suggesting GA-induced cytoprotective autophagy in NSCLC A549 and NCI-H1299 cells. However, IRE1α knockdown failed to increase GA-induced cell proliferative inhibition and apoptosis. This may be owing to the IRE1α induced not only autophagy but apoptosis as well, and the prodeath apoptosis effect of IRE1α potentially balanced prosurvival autophagy [41]. Moreover, the JNK/c-jun pathway could potentially contribute to autophagy instead of apoptosis in A549 cells [58].

**Figure 10:**
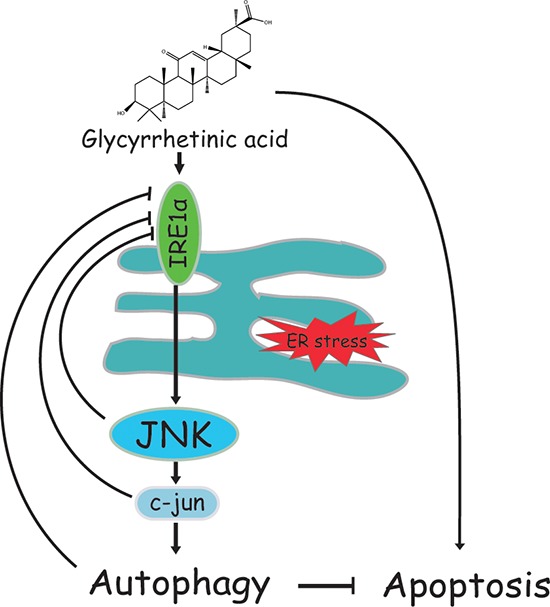
Schematic of GA-induced autophagy in NSCLC cells GA activates autophagy in NSCLC A549 and NCI-H1299 cells by activating the IRE1α-JNK/c-jun pathway. Autophagy or JNK inhibition enhances GA-induced proliferative inhibition, apoptosis, and ER stress.

In conclusion, we demonstrated that GA induced autophagy in NSCLC A549 and NCI-H1299 cells by activation of the IRE1α-JNK/c-jun cascade and that suppression of autophagy remarkably increases GA-induced cell proliferative inhibition, apoptosis, and ER stress (Figure 10). This finding further illustrates the correlation of ER stress, JNK/c-jun, and autophagy. Combined treatment of an autophagy inhibitor (*e.g*., CQ) and GA or GA-contained prescriptions shows potential as a strategy for NSCLC therapy.

## MATERIALS AND METHODS

### Chemicals and antibodies

GA was acquired from the National Institutes for Food and Drug Control in Shenzhen, Guangdong, China. 3-(4,5-dimethylthiazol-2-yl)-2, 5-Diphenyltetrazolium bromide (MTT), SP600125, chloroquine (CQ), and dimethyl sulfoxide (DMSO) were obtained from Sigma (St. Louis, MO, USA). PD98059 and 4′,6-diamidino-2-phenylindole (DAPI) were purchased from Beyotime Biotechnology Corporation–Shanghai (Shanghai, China). RPMI1640 medium, fetal bovine serum (FBS), penicillin, streptomycin, and phosphate-buffered saline (PBS) were purchased from Gibco Life Technologies (Grand Island, NY, USA). Hoechst 33342 was obtained from Molecular Probes (Grand Island, USA). Primary antibodies, including poly (ADP-ribose) polymerase (PARP), Beclin1, ATG5, ATG7, LC3, p-ERK (Thr202/Tyr204), ERK, p-Akt (Ser473), Akt, p-mTOR (Ser2448), mTOR, p-P70 S6k (Ser371), P70 S6K, p-4E-BP1 (Thr37/46), 4E-BP1, p-ULK1 (757), ULK1, p-JNK (Thr183/Tyr185), JNK, p-c-jun (ser63), c-jun, IRE1α, XBP-1s, Bip, and Chop, together with GAPDH antibodies and secondary antibodies, were purchased from Cell Signaling Technology, Inc. (Beverly, MA, USA).

### Cell culture

A549 and NCI-H1299 cells were acquired from American Type Culture Collection (ATCC, Rockville, MD, USA) and cultured in RPMI 1640 medium supplemented with 10% (v/v) FBS and 1% (v/v) antibiotics (100 U/mL penicillin, 100 μg/mL streptomycin). The cells were maintained in an incubator set to 37°C with 5% CO_2_.

### Cell viability assay

The anti-proliferative effect of GA was determined through MTT detection as described in a previous study [59]. Cells were seeded in a 96-well plate at a density of 4–6 × 10^3^ cells/well and cultured to about 70%–80%. Cells were treated with the indicated concentrations of the test compounds. Fresh medium with 1 mg/mL MTT was added to the wells, and the plate was incubated for 4 h at 37°C. The medium was discarded, and 100 μL of DMSO was added to the wells to dissolve the violet formazan crystals. The absorbance of the solution at 570 nm was recorded with a microplate reader (Perkin Elmer, 1420 Multilabel Counter Victor3, Wellesley, MA, USA).

### Clone formation assay

Cells were seeded at a density of 400 cells/well in six-well plates. After attachment, the cells were treated with the indicated concentrations of GA for 24 h. The medium was discarded, and the cells were cultured for another 14 d in fresh medium until visible colonies were observed. The cells were then washed with PBS and subsequently fixed using 4% paraformaldehyde (PFA) for 30 min. Crystal violet staining solution (Beyotime Institute of Biotechnology, China) was used to stain the cells for 30 min. The dye was then washed with PBS, and cell images were captured using an ordinary NIKON camera.

### Annexin V/PI staining assay

After treatment with various GA concentrations for 24 h, the cells were trypsinized, washed, and collected. Apoptotic cells were detected using an Annexin V-FITC kit (Beyotime Biotechnology Corporation, Shanghai, China) in accordance with the protocol provided by the manufacturer. A total of 10,000 cells were subsequently collected and analyzed using a flow cytometer (FACS-Canto, BD Bioscience, USA).

### Hoechst 33342 staining assay

Cells were exposed to various GA concentrations for 24 h, washed with PBS, and fixed with 4% formaldehyde for 30 min. The cells were then stained with 1 μg/mL Hoechst 33342 for 30 min. After PBS washing, nuclei were observed using In Cell Analyzer 2000 (GE Healthcare, Little Chalfont, UK), and cell images were obtained.

### Western blot analysis

After GA treatment, protein expression levels were evaluated by Western blot. The proteins were initially lysed with a radioimmunoprecipitation lysis buffer containing 1% phosphatase inhibitor, phenylmethanesulfonyl fluoride, and 1% protease inhibitor cocktail for 25 min. The protein concentrations of the lysates were then examined using BCA™ Protein Assay Kit (Pierce, Rockford, IL, USA). Equal quantities of proteins were subjected to sodium dodecyl sulfate-polyacrylamide gel electrophoresis, transferred onto polyvinylidene fluoride membranes, and blocked with 5% non-fat dried milk for 1 h at 37°C. The membranes were probed with specific primary antibodies overnight at 4°C and then incubated with corresponding secondary antibodies for 1 h at 37°C. The specific protein bands were visualized with an ECL advanced Western blot analysis detection kit (BD Biosciences, Bedford, MA, USA). Equal protein loading was verified by probing with anti-GAPDH antibodies.

### Caspase-3/7 activity assay

Caspase activity assay kits (Cell Signaling Technology, Inc., Beverly, MA, USA) were used to detect caspase activities in accordance with the manufacturer's instructions. Cells were plated into 96-well plates and cultured for about 24 h. Cells were then incubated with different concentrations of GA with or without pretreatment of CQ or specific knockdown of ATG7, JNK, and IRE1α. 200 μL of caspase-3/7 assay reagent was added to each well and incubated in the dark for 1 h. Luminescence was detected using a microplate reader (Perkin Elmer, 1420 Multilabel Counter Victor3, Wellesley, MA, USA).

### Transfection

The specific target sequences of ATG7 are as follows: sense 5′-GGUCAAAGGACGAAGAUAATT-3′, antisense 5′-UUAUCUUCGUCCUUUGACCTT-3′; Beclin1: sense 5′-GGAGCCAUUUAUUGAAACUTT-3′, antisense 5′-AGUUUCAAUAAAUGGCUCCTT-3′; ATG5: sense 5′-GACGUUGGUAACUGACAAATT-3′, antisense 5′-UUUGUCAGUUACCAACGUCTT-3′; JNK1: sense 5′-GCUCAGGAGCUCAAGGAAUTT-3′, antisense 5′-AUUCCUUGAGCUCCUGAGCTT-3′; JNK2: sense 5′-CCAGCAGCUGAAACCAAUUTT-3′, antisense 5′-AAUUGGUUUCAGCUGCUGGTT-3′; c-jun: sense 5′-GGAACAGGUGGCACAGCUUTT-3′, antisense 5′-AAGCUGUGCCACCUGUUCCTT-3′; IRE1α: sense 5′-CUCCGAGCCAUGAGAAAUATT-3′, antisense 5′-UAUUUCUCAUGGCUCGGAGTT-3′; negative control: sense 5′-UUCUCCGAACGUGUCACGUTT-3′, antisense 5′-ACGUGACACGUUCGGAGAATT-3′ (synthesized by GenePharma, Shanghai, China). GFP-LC3 (supplied by Toren Finkel, Addgene plasmid #24920) and mRFP-GFP-LC3 (supplied by Tamotsu Yoshimori, Addgene plasmid #21074) plasmids were obtained from Addgene [60, 61]. Cells were seeded overnight and then transfected with siRNA or DNA plasmids using Lipofectamine™ 2000 transfection reagent (Invitrogen Corp., Carlsbad, CA, USA) in accordance with the manufacturer's instructions. The medium was changed to GA, and cells were incubated for another 24 h. Proliferative inhibition was detected by MTT assay, caspase-3/7 activation was studied using a commercial assay kit, and protein expression levels were determined by Western blot.

### Immunofluorescence staining

After seeding in a 96-well plate overnight, A549 cells were transiently transfected with GFP-LC3 or mRFP-GFP-LC3 plasmids for 24 h with or without subsequent 24 h transfection with specific siRNAs (*i.e*., scrambled, ATG 7, JNK, and IRE1α siRNAs). The cells were incubated with the indicated compounds for 24 h. After incubation, the cells were fixed with 4% PFA, washed with PBS, and incubated with DAPI for 15 min. Immunofluorescence images were obtained using In Cell Analyzer 2000.

### Statistical analysis

The mean ± standard deviation was determined for each group. Statistical analysis was performed using ANOVA and Tukey's test. Each experiment had at least three independent replicates, and significant differences were indicated as *P < 0.05 and **P < 0.01.

## SUPPLEMENTARY FIGURES


