# Supplementary material for: Glycyrrhetinic acid induces cytoprotective autophagy via the inositol-requiring enzyme 1α-c-Jun N-terminal kinase cascade in non-small cell lung cancer cells
Source: Oncotarget. 2015 Nov 3;6(41):43911–26. doi: 10.18632/oncotarget.6084 (PMC4791276; doi:10.18632/oncotarget.6084)
Supplement: Supplementary file 1 [file oncotarget-06-43911-s001.pdf]

SUPPLEMENTARY FIGURES

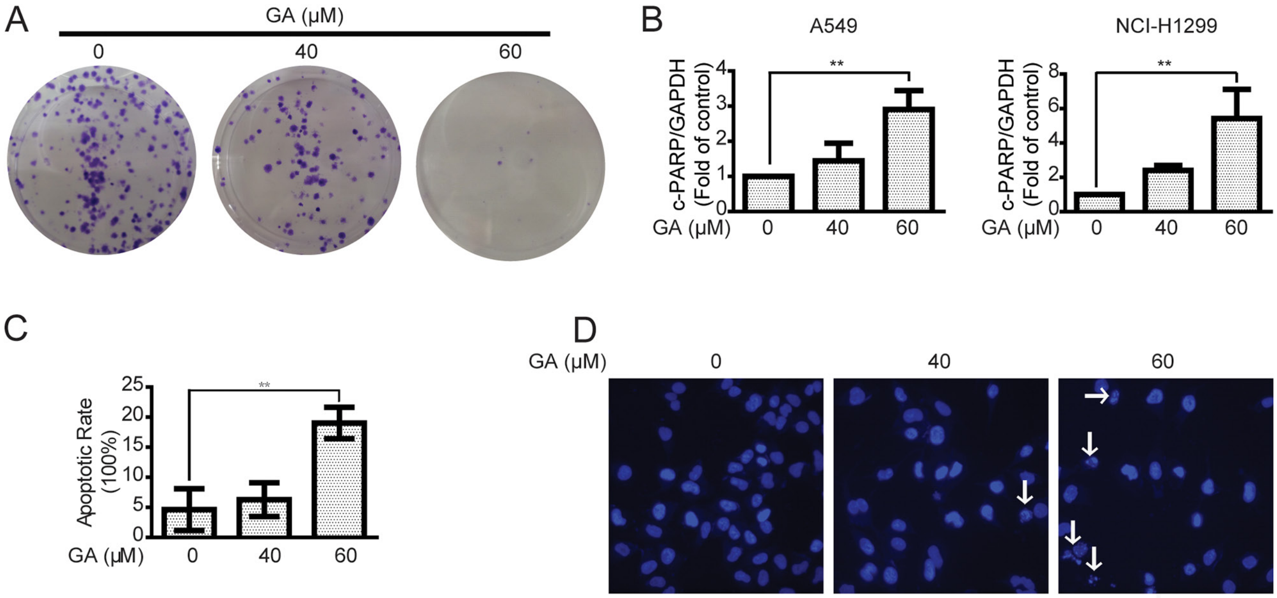

Supplementary Figure S1: GA induces cell colony formation inhibition and apoptosis in A549 cells. A. A549 cells were seeded in a six-well plate at a density of 400 cells per well and then cultured overnight. The cells were then treated with different concentrations of GA for 24 h, cultured in fresh medium for about 14 d, and subsequently fixed and stained with 4% PFA and crystal violet. Representative images were presented. **B.** The statistical analysis of Figure 1C–1D. $P < 0.05$ and $^{**}P < 0.01$. **C.** After GA treatment for 24 h, apoptotic A549 cells were stained with Annexin V/PI and analyzed by flow cytometry. $^{*}P < 0.05$ and $^{**}P < 0.01$. **D.** A549 cells were treated with the indicated concentrations of GA for 24 h, and the nuclei were stained with DAPI. Typical images were presented.

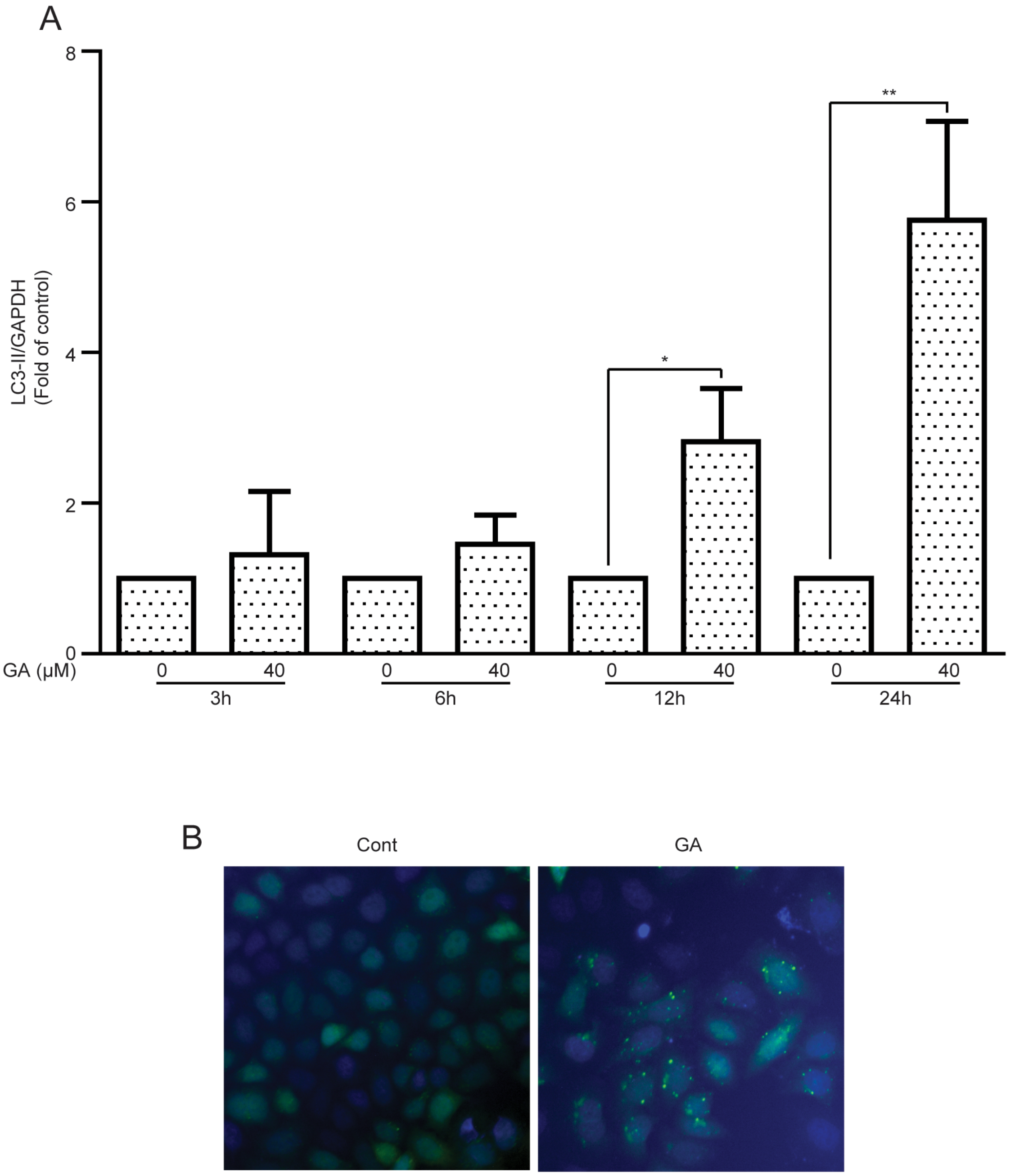

Supplementary Figure S2: GA increases LC3-II protein level and GFP-LC3 punta formation. A. Statistical analysis related to Figure 2C. $P < 0.05$ and $^{**}P < 0.01$. **B.** After treatment with GA (40 μM) for 24 h, the GFP-LC3 punta formation was observed in GFP-LC3 stably expressing HeLa cells by using IN Cell Analyzer 2000, and nuclear staining was performed using DAPI.

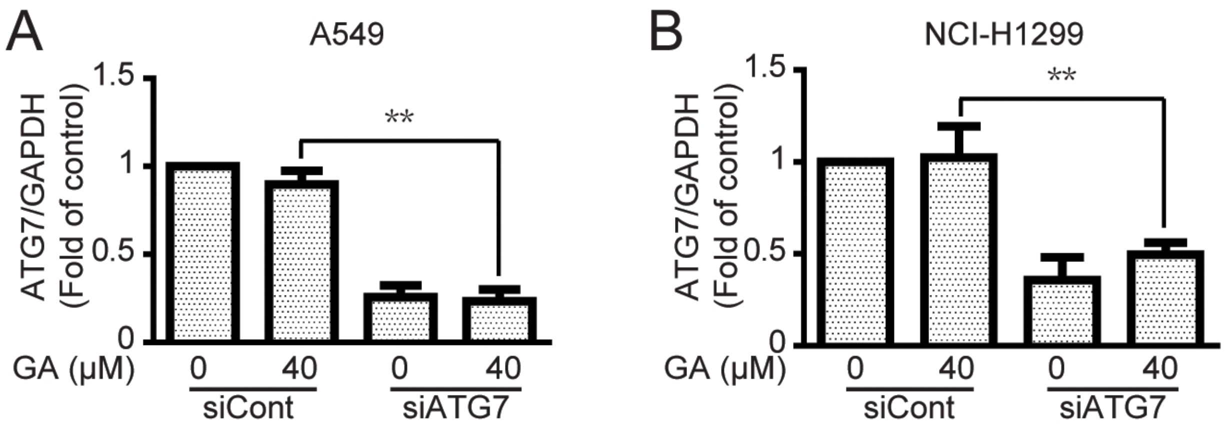

Supplementary Figure S3: ATG7 protein is silenced by siRNA. A–B. Statistical analysis related to Figure 4A–4B. *P* < 0.05 and ***P* < 0.01.

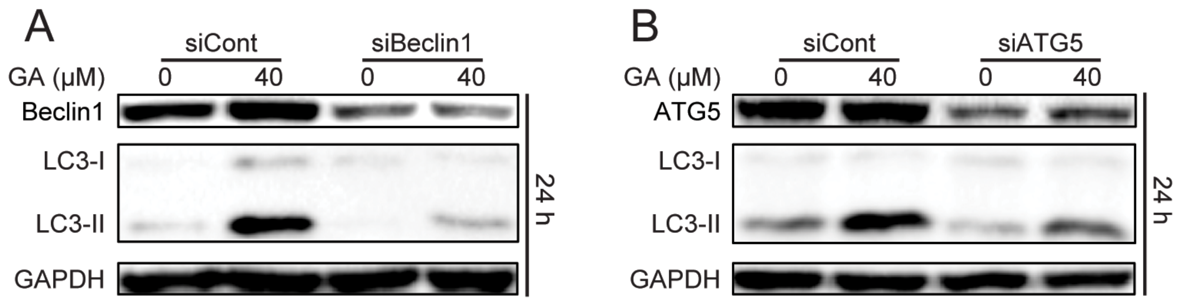

Supplementary Figure S4: GA-induced autophagy is decreased after knockdown of ATG5 or Beclin1 in A549 cells. A–B. After transient transfection with Beclin1 or ATG5 siRNA for 24 h, A549 cells were treated with 40 μM GA for 24 h, and the indicated protein expression was evaluated by Western blot analysis.

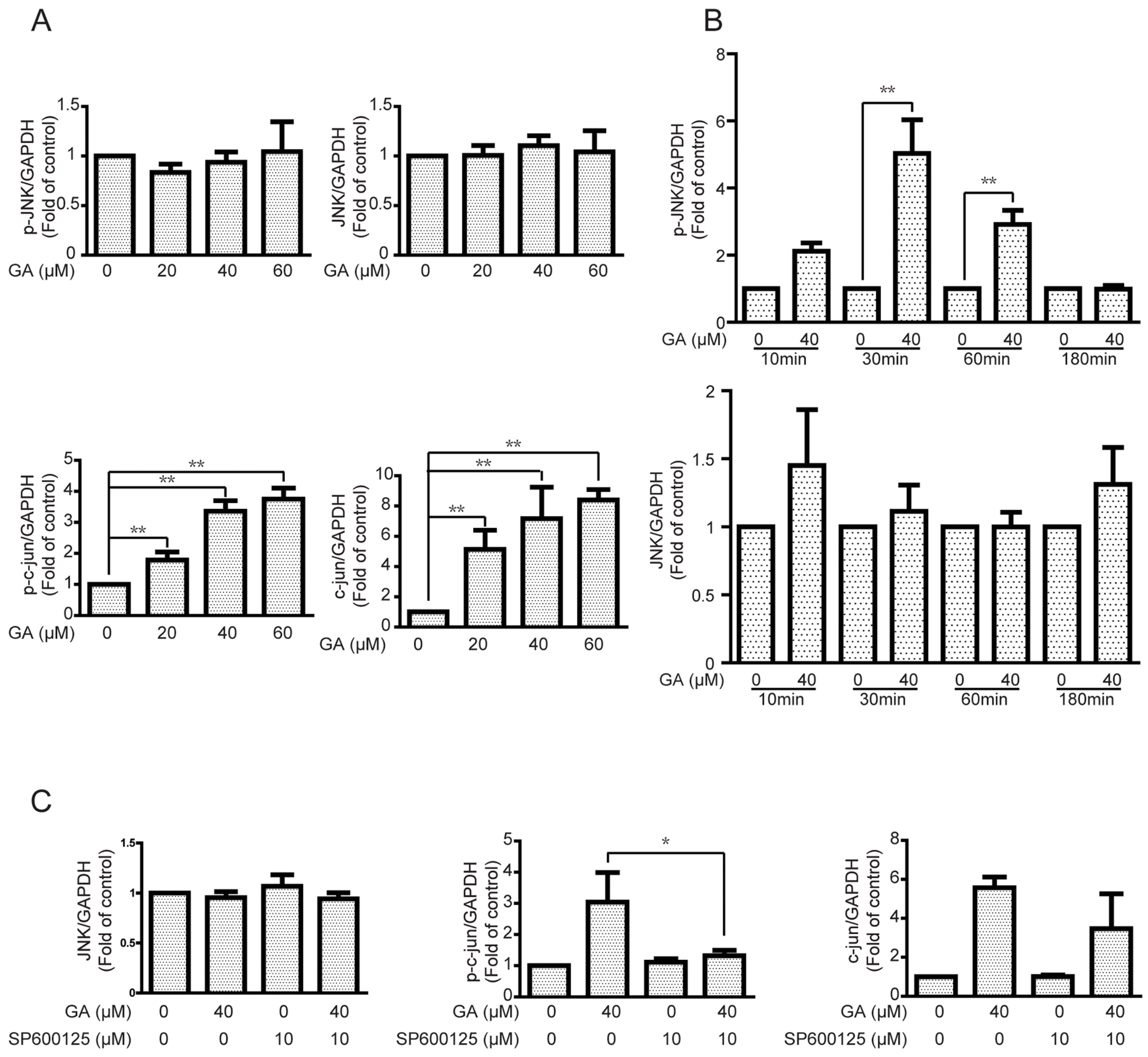

Supplementary Figure S5: GA induces autophagy by activation of the JNK/c-jun pathway. A–C. Statistical analysis related to Figure 5A–5C. $P < 0.05$ and $**P < 0.01$.

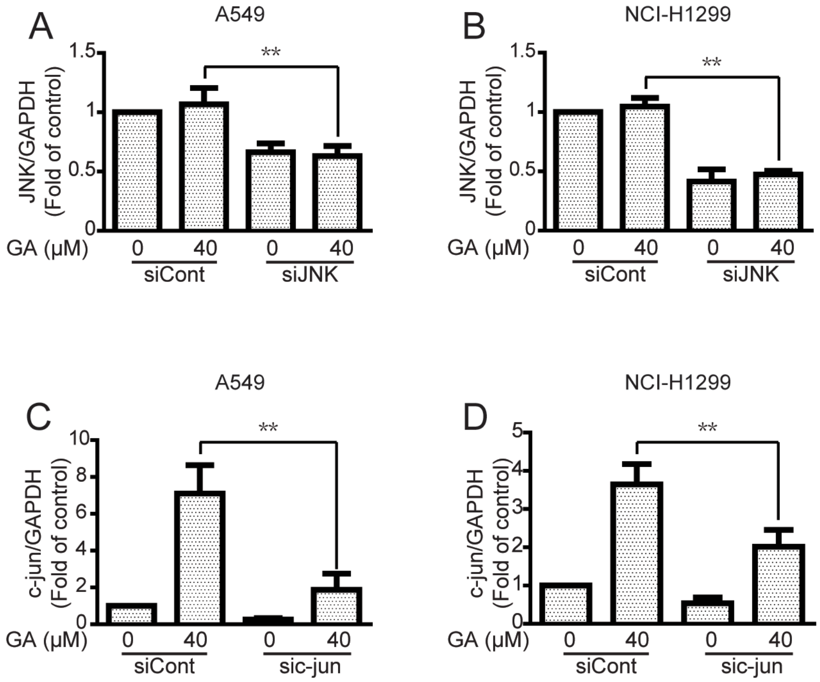

Supplementary Figure S6: JNK and c-jun proteins are silenced by siRNA in A549 and NCI-H1299 cells. A–D. Statistical analysis related to Figure 6A–6B and 6D–6E. $P < 0.05$ and $**P < 0.01$.

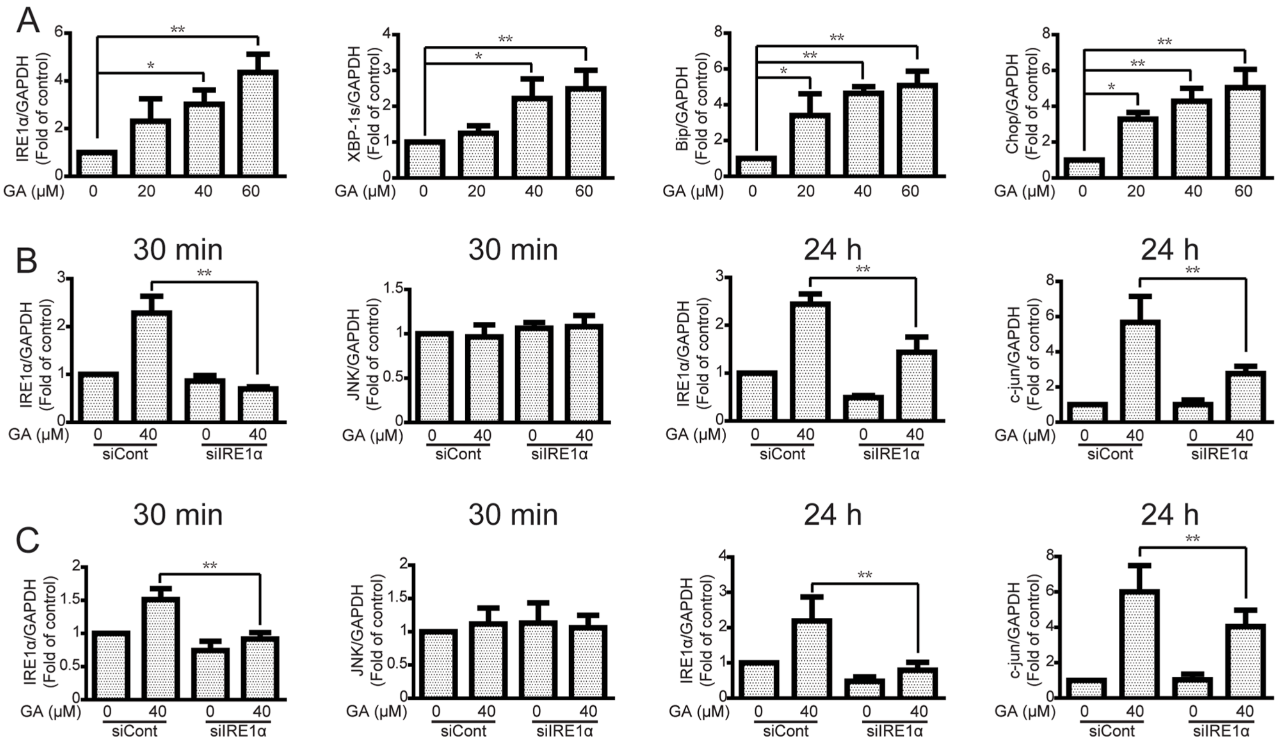

Supplementary Figure S7: GA induces autophagy by activation of IRE1α-JNK/c-jun pathway in A549 and NCI-H1299 cells. A–C. Statistical analysis related to Figure 7A and 7C–7D. $P < 0.05$ and $**P < 0.01$.

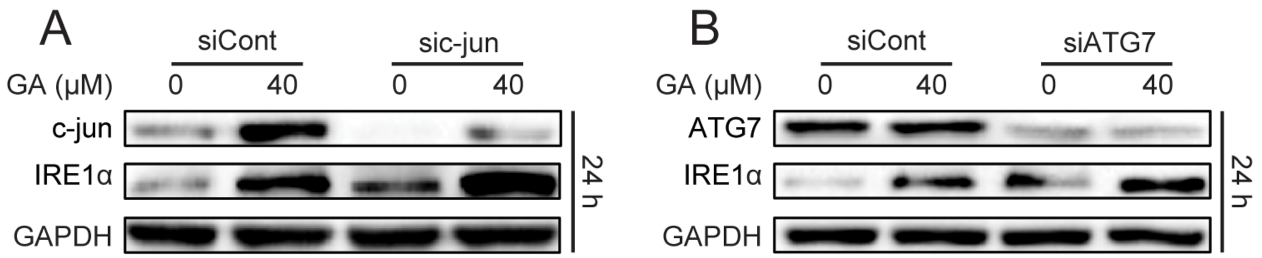

Supplementary Figure S8: GA-induced ER stress is increased after silence of c-jun or ATG7. A–B. After transient transfection with c-jun or ATG7 siRNA for 24 h, A549 cells were treated with GA (40 μM) for 24 h, and the indicated protein expression level was evaluated by Western blot analysis.

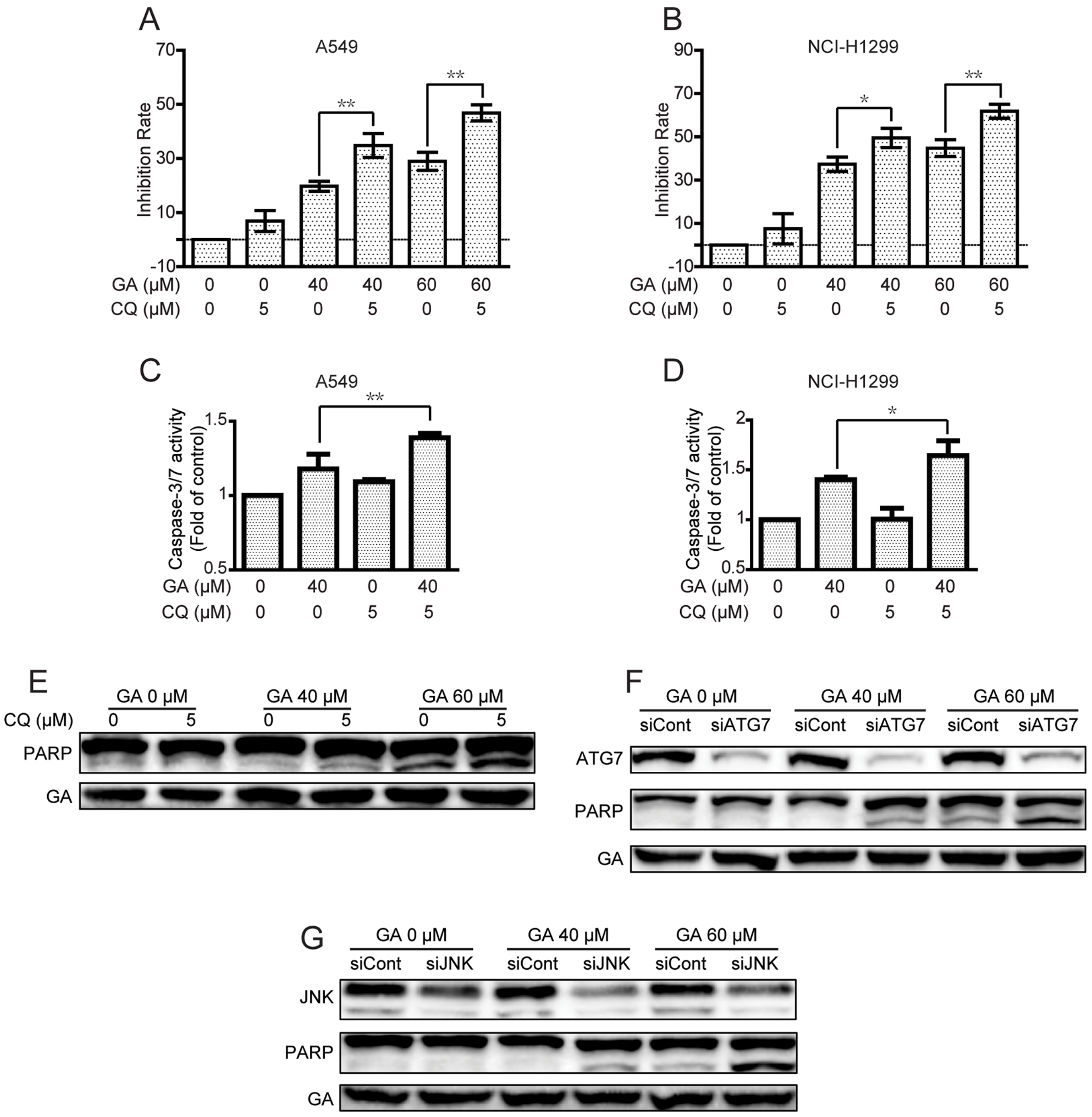

Supplementary Figure S9: GA-induced cell proliferative inhibition and apoptosis are increased after suppression of autophagy or JNK in A549 and NCI-H1299 cells. **A–D.** A549 and NCI-H1299 cells were cultured in indicated concentrations of GA for 24 h with or without CQ pretreatment (5 μM, 1 h). Cell proliferative inhibition was examined by MTT assay, and caspase-3/7 activation was detected using a commercial assay kit $^*P < 0.05$ and $^{**}P < 0.01$. **E.** A549 cells were GA-treated for 24 h with or without CQ pretreatment (5 μM, 1 h). Protein expression was detected by Western blot analysis. **F–G.** A549 cells were transiently transfected with ATG 7 or JNK siRNA and then cultured with indicated concentrations of GA for 24 h. Western blot analysis was used to detect PARP expression levels.

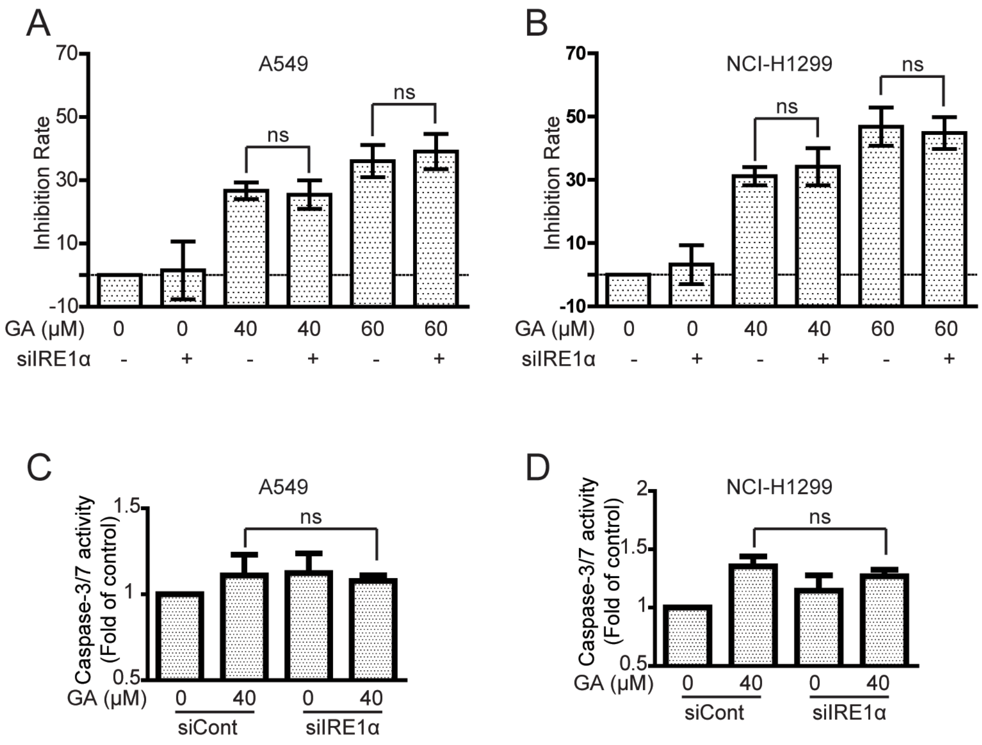

Supplementary Figure S10: GA-induced cell proliferative inhibition and apoptosis did not markedly change after knockdown of IRE1α in A549 and NCI-H1299 cells. A–D. A549 and NCI-H1299 cells were GA-treated for 24 h with or without pretreatment of IRE1α siRNA. Cell viability was evaluated by MTT assay, and caspase-3/7 activation was detected using a commercial assay kit. "No statistical difference" was denoted by "ns."

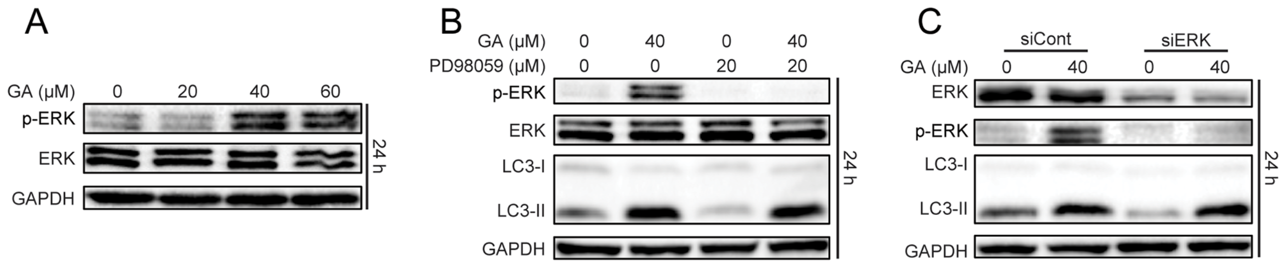

Supplementary Figure S11: GA-induced autophagy is independent of ERK pathway in A549 cells. A. A549 cells were treated with various concentrations of GA for 24 h, and protein expression was evaluated by Western blot assay. **B.** After pretreatment with the ERK pathway inhibitor PD98059 (20 μM, 1 h), A549 cells were GA-treated for 24 h, and protein expression was determined by Western blot analysis. **C.** After knockdown of ERK by siRNA, A549 cells were cultured for 24 h and then treated with GA (40 μM) GA for 24 h. The protein change was detected by Western blot analysis.

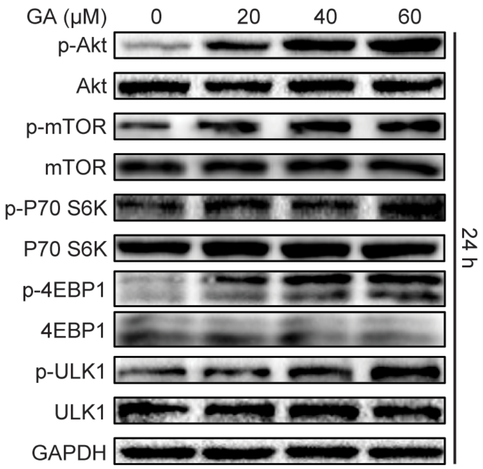

Supplementary Figure S12: GA activates the Akt/mTOR cascade. A549 cells were treated with indicated concentrations of GA for 24 h, and the Akt/mTOR pathway-related proteins were determined by Western blot analysis.

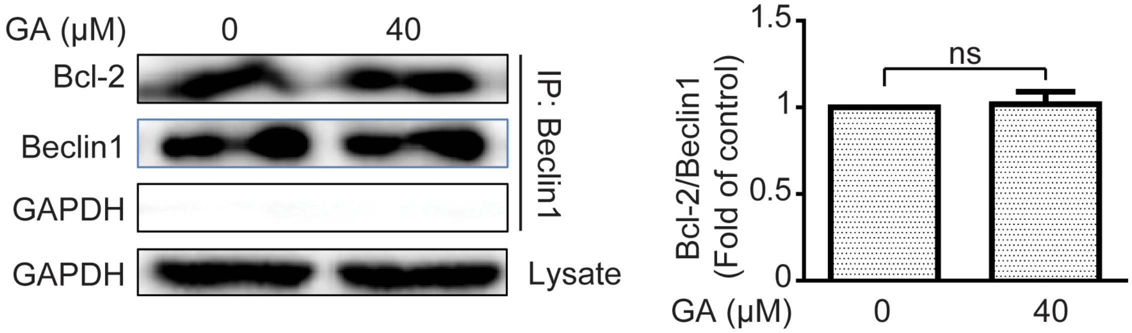

Supplementary Figure S13: Bcl-2/Beclin1 interaction activity is not affected by GA treatment. A549 cells were treated with GA (40 μM) GA for 24 h, and cell lysates were prepared and subjected to immunoprecipitation with Beclin 1. Immunoblotting with Bcl-2 and GAPDH followed. The samples were blotted with anti-Beclin 1 as a control. "No statistical difference" was denoted by "ns."
